# Fructose Levels Are Markedly Elevated in Cerebrospinal Fluid Compared to Plasma in Pregnant Women

**DOI:** 10.1371/journal.pone.0128582

**Published:** 2015-06-02

**Authors:** Janice J. Hwang, Andrea Johnson, Gary Cline, Renata Belfort-DeAguiar, Denis Snegovskikh, Babar Khokhar, Christina S. Han, Robert S. Sherwin

**Affiliations:** 1 Yale University School of Medicine, Division of Endocrinology, New Haven, Connecticut, United States of America; 2 Yale University School of Medicine, Department of Obstetrics and Gynecology, New Haven, Connecticut, United States of America; 3 Yale University School of Medicine, Department of Anesthesia, New Haven, Connecticut, United States of America; 4 Yale University School of Medicine, Department of Neurology, New Haven, Connecticut, United States of America; University of Maryland, UNITED STATES

## Abstract

**Background:**

Fructose, unlike glucose, promotes feeding behavior in rodents and its ingestion exerts differential effects in the human brain. However, plasma fructose is typically 1/1000th of glucose levels and it is unclear to what extent fructose crosses the blood-brain barrier. We investigated whether local endogenous central nervous system (CNS) fructose production from glucose via the polyol pathway (glucose→sorbitol→fructose) contributes to brain exposure to fructose.

**Methods:**

In this observational study, fasting glucose, sorbitol and fructose concentrations were measured using gas-chromatography-liquid mass spectroscopy in cerebrospinal fluid (CSF), maternal plasma, and venous cord blood collected from 25 pregnant women (6 lean, 10 overweight/obese, and 9 T2DM/gestational DM) undergoing spinal anesthesia and elective cesarean section.

**Results:**

As expected, CSF glucose was ~60% of plasma glucose levels. In contrast, fructose was nearly 20-fold higher in CSF than in plasma (p < 0.001), and CSF sorbitol was ~9-times higher than plasma levels (p < 0.001). Moreover, CSF fructose correlated positively with CSF glucose (ρ 0.45, p = 0.02) and sorbitol levels (ρ 0.75, p < 0.001). Cord blood sorbitol was also ~7-fold higher than maternal plasma sorbitol levels (p = 0.001). There were no differences in plasma, CSF, and cord blood glucose, fructose, or sorbitol levels between groups.

**Conclusions:**

These data raise the possibility that fructose may be produced endogenously in the human brain and that the effects of fructose in the human brain and placenta may extend beyond its dietary consumption.

## Introduction

The rising incidence of type 2 diabetes (T2DM) and obesity has paralleled the increased consumption of fructose, typically in the form of sucrose and high fructose sweeteners, which have been associated with numerous adverse effects including weight gain, insulin resistance, and cognitive decline [1,2]. In isolation, fructose and glucose have opposite effects in the central nervous system (CNS). In rats, intraventricular glucose infusion into the brain decreases feeding, whereas infusion of fructose promotes feeding [3]. Moreover, in healthy lean adults, ingestion of fructose leads to a different pattern of cerebral blood flow in brain regions associated with appetite and reward compared to ingestion of the same amount of glucose [4]. Given that glucose is delivered to the brain via passage across the blood-brain barrier (BBB) [5,6], most studies have generally assumed that the CNS effects of fructose are mediated by peripheral fructose crossing the BBB as well [7]. However, plasma fructose levels are exceedingly low due to its efficient metabolism by the liver. Thus, whether peripheral fructose crosses the blood-brain barrier in levels sufficient to generate its CNS effects remains uncertain.

Fructose can be converted from glucose via the polyol pathway (glucose → sorbitol → fructose), an alternate glucose pathway that bypasses the control points of hexokinase and phosphofructokinase [8,9]. Both aldose reductase (which reduces glucose to sorbitol) and sorbitol dehydrogenase (which oxidizes sorbitol to fructose), are present throughout the body including in the brain [10,11] and placenta [8,12]. In rats, 3-fluoro-3-deoxy-D-glucose infusion increases sorbitol and fructose in the brain as measured by NMR spectroscopy, with reduced flux following administration of aldose reductase inhibitors [10]. In dogs, peripheral glucose infusion increases levels of fructose and sorbitol in the cerebrospinal fluid (CSF) [13,14]. Furthermore, alloxan and growth hormone-induced diabetic dogs have been reported to have 7 and 10-fold higher CSF fructose levels, respectively, compared to non-diabetic animals [15,16]. In humans, CSF fructose levels were first reported to be higher than plasma levels in the 1930’s [17], an observation later confirmed in a cohort of diabetic and non-diabetic individuals [18]. The study, however, was limited by the technical inability to precisely measure fructose, the inclusion of both fasting and non-fasting subjects, and the lack of assessment of diabetes control or type of diabetes [18]. Since then, to our knowledge there have been no human studies using modern assays for fructose and sorbitol to examine whether they are generated within the CNS via glucose flux through the polyol pathway. Furthermore, because aldose reductase is highly expressed in the placenta [8,12], we also sought to evaluate the effects of maternal glucose levels on fetal exposure to sorbitol and fructose by measuring cord venous blood (reflecting blood from placenta to fetus) levels of these metabolites. Thus, we evaluated the presence of polyol pathway activity in the CNS and cord blood in lean, overweight/obese, and T2DM/gestational DM (GDM) women in the late stages of pregnancy, a time of increased maternal energy requirements to meet the increased energy demands of the fetus and placenta.

## Methods

### Participants

25 pregnant women scheduled for elective cesarean section at term participated in this study (Clinical Trial Registration number: NCT 02109094). They were recruited through advertisements in the New Haven area. Women who were lean (pre-pregnancy BMI <25 kg/m2), overweight/obese (pre-pregnancy BMI ≥25 kg/m2), and/or had preexisting T2DM or GDM diagnosed through routine prenatal screening were included. Exclusion criteria included medical disorders other than diabetes, use of medications other than prenatal vitamin, folic acid, or diabetic medications, and obstetrical problems including: preeclampsia, major fetal anomalies, smoking or illicit drug use. The Yale University Human Investigation Committee approved the protocol and all subjects provided written informed consent.

### Study procedures

Participants (6 lean, 10 overweight/obese, and 9 T2DM/GDM) arrived fasting on the day of scheduled, elective cesarean section in the absence of labor. There were no alterations in their prenatal, pre-operative, or intra-operative care with the exception of maternal blood, CSF, and umbilical cord blood collections. Maternal blood was collected at the time of IV insertion. Maternal CSF (approximately 3–5 ml) was collected at the time of spinal/epidural anesthesia before anesthetic/analgesia injection by a qualified anesthesiologist. CSF cell counts were obtained to ensure absence of contamination from blood. Two subjects had >5 RBCs per high-power field in their CSF; however, there were no differences in CSF fructose, glucose, or sorbitol levels and those subjects were included in the analysis. Following infant delivery a sterile segment of umbilical cord was doubly clamped and cord venous blood was drawn prior to delivery of the placenta. Cord venous blood was unable to be obtained from 4 women due to insufficient quantities and clotting.

Plasma and CSF fructose and sorbitol levels were measured using gas chromatography-liquid mass spectrometry. Fructose levels were below detection limits for 5 maternal plasma (3 obese, 1 lean, 1 DM subject) and 4 cord plasma samples (3 obese, 1 lean subject) and were excluded. Glucose levels were measured enzymatically with glucose oxidase (YSI, Yellow Springs, OH, USA).

### Statistical analysis

CSF and plasma fructose and sorbitol levels were not normally distributed and were compared using the independent samples Kruskal-Wallis test. Wilcoxon Signed Rank Test was used for within subject comparisons. Correlations were assessed using Spearman correlation coefficient. Glucose was normally distributed and one-way ANOVA was used for across group comparisons and t-test for post-hoc analysis. Two-tailed probability values are reported. Statistical significance was assumed when p < 0.05. Glucose data are reported as mean ± SE; fructose and sorbitol data are reported as both median and mean. All statistical analyses were performed using SPSS (version 19, IBM).

## Results

As shown in Table 1, there were no age differences between the groups (p = 0.49). Hemoglobin A1C differed across groups (p = 0.03) and was modestly lower in lean pregnant women (5.0% ± 0.1) compared to both diabetic women (HbA1C 5.5% ± 0.1, p = 0.01) and overweight/obese women (HbA1C 5.6% ± 0.2, p = 0.02). No differences were observed in fasting plasma glucose levels between the groups (p = 0.61). Among women with diabetes, 4 were treated with insulin, 2 were diet-controlled and 3 received oral medications. Pre-pregnancy BMI differed between the groups (p = 0.02). Nevertheless, no differences in weight gain occurred during pregnancy between any of the groups (p = 0.40).

**Table 1:** Subject characteristics.

	**All Subjects**	**Lean (n = 6)**	**Obese (n = 10)**	**DM (n = 9)**	P*
**Age (years)**	33.3 ± 1.2	33.5 ± 2.2	31.6 ± 2.0	35.11 ± 2.2	0.49
**Gestational age (wks)**	39.1 ± 0.1	39.1± 0.0	39.2 ± 0.3	39.0 ± 0.3	0.75
**Hb A1C (%)**	5.4 ± 0.1	5.0 ± 0.1	5.6 ± 0.2	5.5 ± 0.1	0.03
**Hb A1C (mmol/mol)**	35.8 ± 1.0	31.1 ± 2.3	37.8 ± 5.6	36.7 ± 4.4	0.03
**Pre-preg BMI (kg/m2)**	31.5 ± 2.7	22.7 ± 0.7	40.4 ± 5.6	27.5 ± 1.9	0.02
**Pregnancy weight gain (kg)**	13.3 ± 1.4	14.6 ± 1.8	10.9 ± 1.9	15.2 ± 3.2	0.35

P values across groups

**Fig 1:**
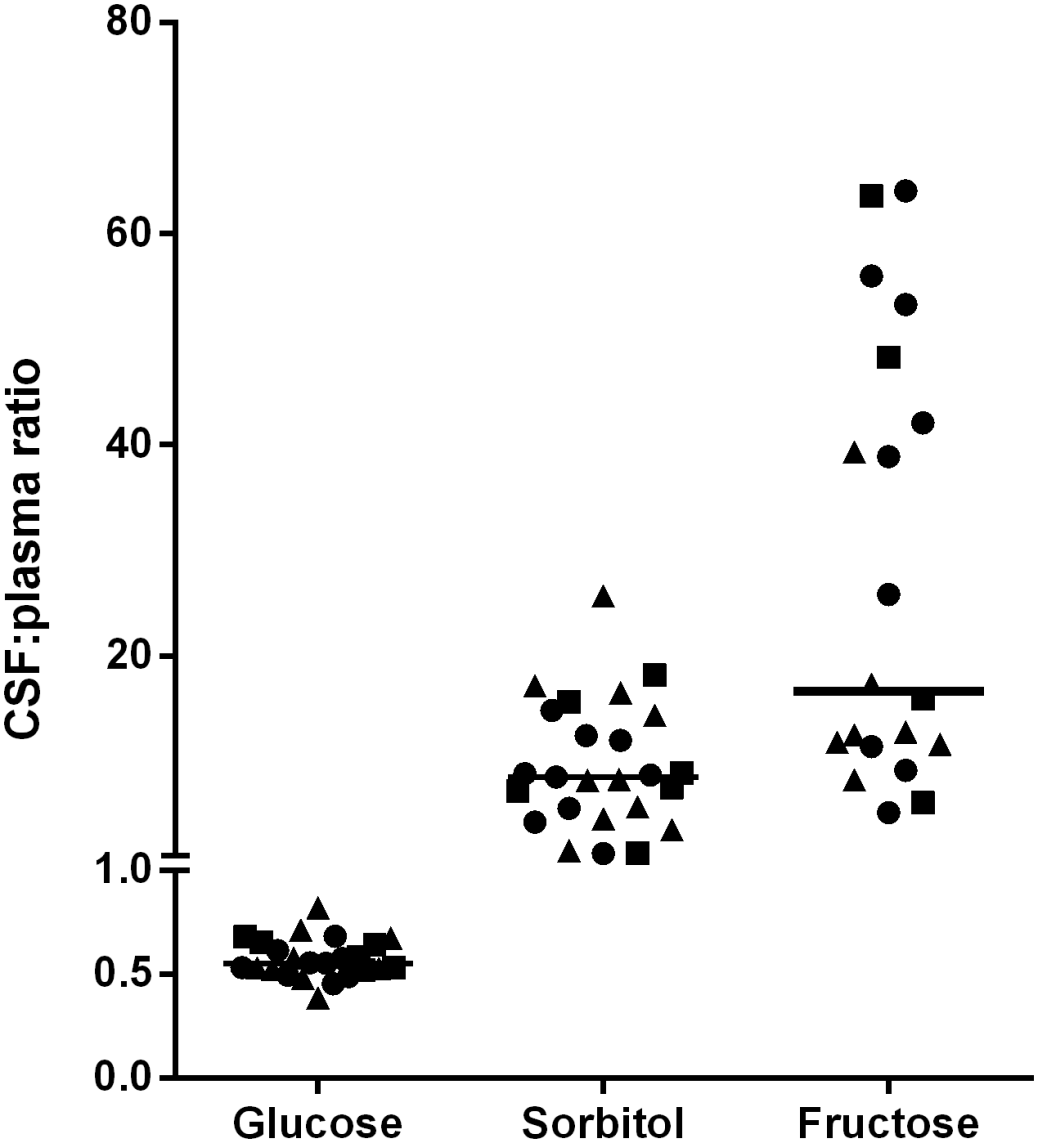
CSF to plasma ratios of glucose, sorbitol, and fructose. Line represents median ratio. Circle = DM; Square = Lean; Triangle = Overweight/Obese

**Fig 2:**
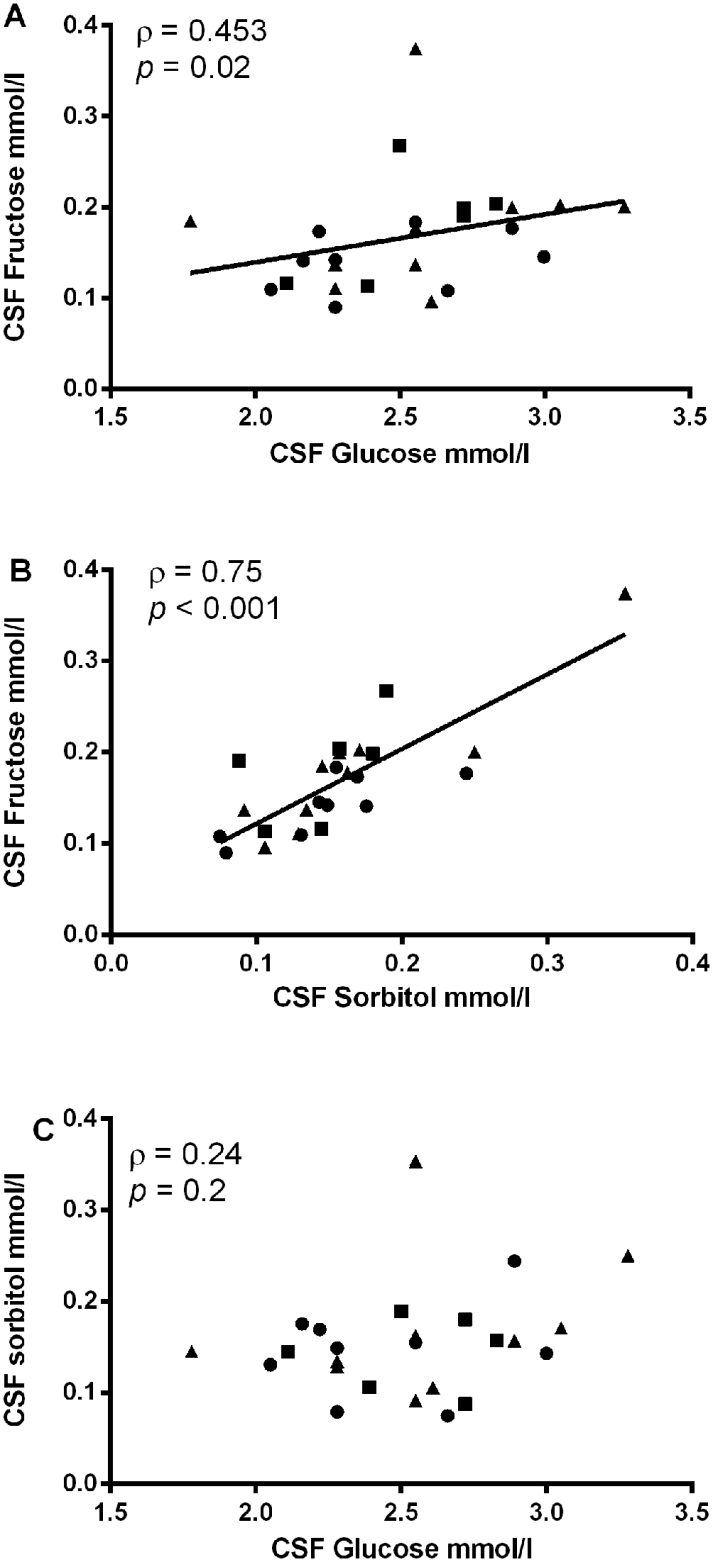
Correlations between (A) CSF fructose and CSF glucose levels; (B) CSF fructose and CSF sorbitol levels; (C) CSF glucose and CSF sorbitol levels. Circle = DM; Square = Lean; Triangle = Overweight/Obese

As shown in Table 2, there were no differences in CSF, plasma, and cord blood levels of glucose, sorbitol or fructose between groups and thus all subjects were analyzed together. Both CSF fructose and CSF sorbitol levels were markedly higher than plasma levels (p < 0.001 for both comparisons). Moreover, as shown in Fig 1, in every subject CSF fructose and sorbitol levels exceeded plasma levels and the median CSF:plasma ratio for fructose was nearly 20 and for sorbitol was ~ 9. As expected, the CSF glucose levels were lower than plasma glucose levels. Despite the narrow range of CSF glucose levels, CSF fructose correlated positively with CSF glucose levels (Fig 2A, ρ 0.45, p = 0.02) as well as with CSF sorbitol levels (Fig 2B, ρ 0.75, p < 0.001). No correlation was detected between CSF glucose and CSF sorbitol levels (Fig 2C, ρ 0.24, p = 0.2).

**Table 2:** Glucose, fructose and sorbitol levels for all subjects.

	**All Subjects**	**Lean (n = 6)**	**Obese (n = 10)**	**DM (n = 9)**	**P** *
**Plasma Glucose**	4.3 ± 0.1	4.3 ± 0.2	4.2 ± 0.2	4.5 ± 0.2	0.61
**CSF glucose**	2.5 ± 0.1	2.5 ± 0.1	2.6 ± 0.1	2.5 ± 0.1	0.65
**CSF:plasma ratio**	0.6 ± 0.04	0.6 ± 0.03	0.7 ± 0.1	0.6 ± 0.02	0.30
**Cord glucose**	3.2 ± 0.3	3.9 ± 0.2	2.5 ± 0.6	3.7 ± 0.2	0.58
**Plasma Fructose**					
** Mean**	0.01 ± 0.001	0.008 ± 0.004	0.012 ± 0.001	0.007 ± 0.002	0.22
** Median (IQR)**	0.009 (0.003, 0.015)	0.006 (0.003, 0.016)	0.01 (0.01, 0.015)	0.003 (0.003, 0.014)	
**CSF Fructose**					
** Mean**	0.17 ± 0.01	0.18 ± 0.02	0.18 ± 0.02	0.14 ± 0.01	0.16
** Median (IQR)**	0.17 (0.11, 0.19)	0.19 (0.12, 0.21)	0.18 (0.13, 0.20)	0.14 (0.11, 0.18)	
**CSF:plasma ratio**					
** Mean**	27 ± 5	34 ± 14	16 ± 4	34 ± 7	0.47
** Median (IQR)**	17 (11, 47)	32 (9, 60)	13 (12, 17)	39 (10, 55)	
**Cord fructose**					
** Mean**	0.009 ± 0.001	0.01 ± 0.004	0.008 ± 0.001	0.009 ± 0.001	0.65
** Median (IQR)**	0.009 (0.006, 0.01)	0.01 (0.005, 0.01)	0.007 (0.006, 0.01)	0.009 (0.007, 0.01)	
**Plasma sorbitol**					
** Mean**	0.02 ± 0.00	0.03 ± 0.01	0.02 ± 0.01	0.02 ± 0.00	0.97
** Median (IQR)**	0.02 (0.01, 0.03)	0.02 (0.01, 0.04)	0.02 (0.01, 0.03)	0.02 (0.015, 0.02)	
**CSF sorbitol**					
** Mean**	0.17 ± 0.02	0.15 ± 0.02	0.17 ± 0.02	0.14 ± 0.02	0.89
** Median (IQR)**	0.15 (0.12, 0.17)	0.15 (0.10, 0.18)	0.15 (0.12, 0.19)	0.15 (0.11, 0.17)	
**CSF:plasma ratio**					
** Mean**	9.6 ± 1	9.8 ± 6.1	10.5 ± 2.4	8.5 ±4.3	0.92
** Median (IQR)**	8.5 (5, 15)	8.2 (6, 16)	8.2 (4, 17)	8.7 (5, 12)	
**Cord sorbitol**					
** Mean**	0.13 ± 0.02	0.08 ± 0.03	0.17 ± 0.04	0.12 ± 0.02	0.48
** Median (IQR)**	0.12 (0.08, 0.16)	0.11 (0.00, 0.13)	0.14 (0.09, 0.25)	0.13 (0.08, 0.16)	

* P values across groups; Glucose, fructose, and sorbitol in mmol/l; Unless otherwise noted, data presented as mean ± SE

**Fig 3:**
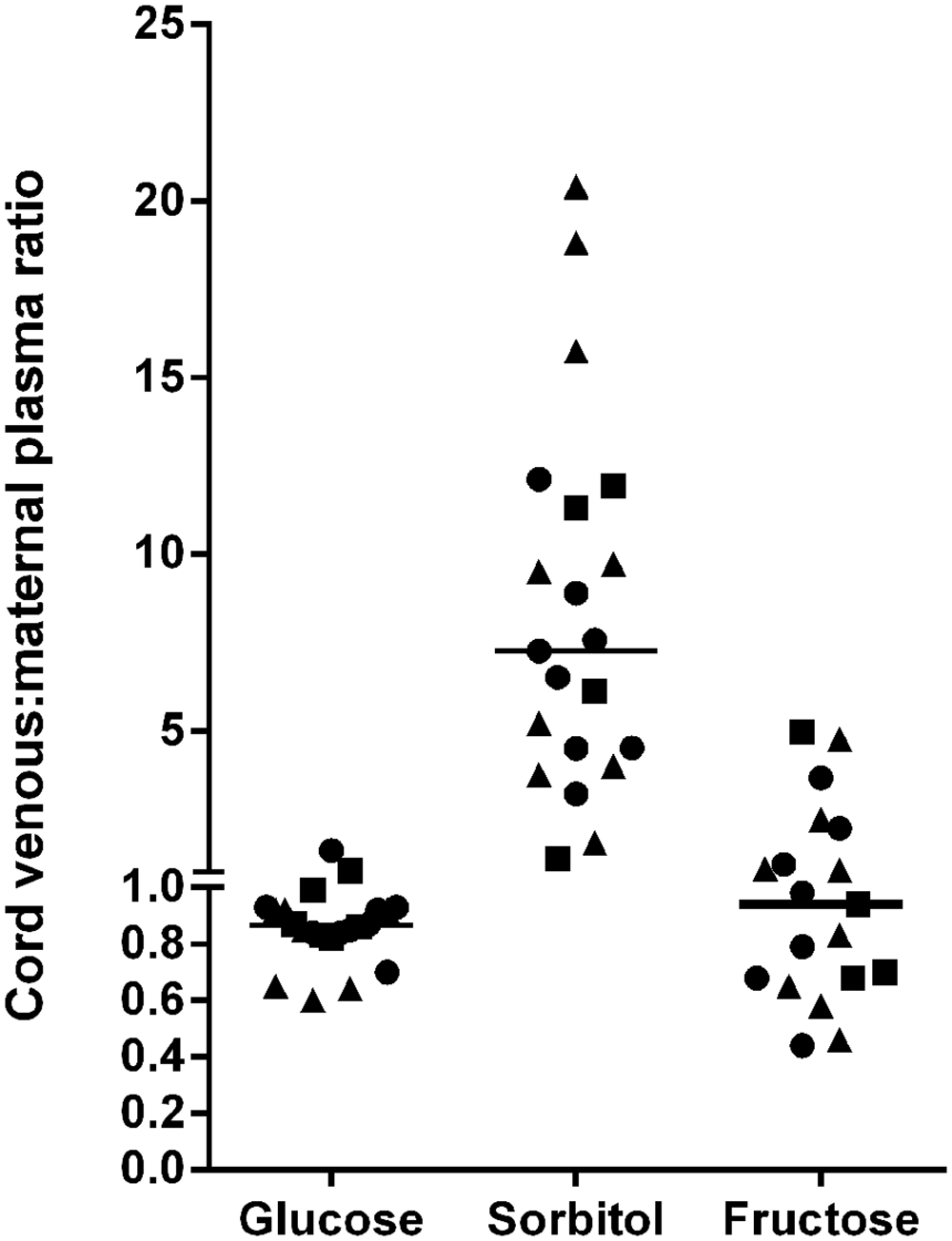
Cord venous blood to maternal plasma ratios of glucose, sorbitol, and fructose. Line represents median ratio. Circle = DM; Square = Lean; Triangle = Overweight/Obese

As shown in Table 2 and Fig 3, mean cord blood glucose levels were approximately 80–90% of maternal plasma levels and correlated positively with maternal plasma glucose levels (ρ 0.440, p = 0.04). Cord blood sorbitol levels were ~7-fold higher than maternal plasma levels (p < 0.001); however, there were no significant differences between cord blood and maternal fructose levels (p = 0.9).

## Discussion

The role of fructose in the CNS remains poorly understood, particularly in humans. In the current study we observed that CSF levels of fructose in pregnant women after an overnight fast are nearly 20-fold higher than in plasma, and as a result, while plasma fructose levels are only 1/1000th of plasma glucose levels, CSF fructose is approximately 6–7% of the levels of glucose in CSF. In addition, CSF fructose correlated positively with both CSF glucose and CSF sorbitol levels, suggesting that fructose within CSF may be driven in large part by endogenous CNS production via the polyol pathway. These findings using modern, more accurate gas chromatography-mass spectroscopy methods are consistent with much earlier studies in animals and non-pregnant humans [10,15–18]. Although there have been reports in rodents that fructose can cross from the periphery into the CNS [4,7], the extent to which this occurs remains uncertain. It is noteworthy in this regard that peripheral infusion of fructose in rodents in doses that increase plasma fructose levels 140-fold only produced a 2.5 fold-increase in ventromedial hypothalamus microdialysate levels [4]. These observations suggest that fructose transport across the BBB is very limited and that the high CSF levels of fructose we observed in humans are likely mainly the result of endogenous CNS production rather than transport across BBB.

The presence of endogenously generated fructose in the brain may have implications for feeding behavior given recent evidence that intraventricular fructose, unlike glucose, promotes feeding in rodents [3]. Aldose reductase activity is amplified in high glucose states [8,9]; thus, there may be increased flux through the pathway when glucose levels rise such as after eating. The relatively lower concentrations of fructose in the CNS compared to glucose may thus make it a more sensitive neuronal signal for carbohydrate ingestion and could have direct or indirect effects to modulate feeding behavior. Interestingly, in Drosophila, ingestion of glucose (or any nutritious carbohydrate) results in increased hemolymph fructose levels, which in turn act on a fructose-specific nutrient sensor in the brain to either stimulate or suppress feeding depending on the satiety state of the fly [19]. To our knowledge, the effect of polyol pathway activity on feeding behavior has not been studied in higher organisms such as rodents or humans. So, it remains uncertain whether or how the observations in Drosophila translate into mammals and humans.

In the periphery, excess polyol pathway activity has been associated with increased osmotic stress [20]. Furthermore, aldose reductase and sorbitol dehydrogenase enzymatic activity also oxidizes NADPH and reduces NAD+, respectively, and excess flux through the polyol pathway has been shown to increase intracellular oxidative stress. This has been implicated in many diabetes related complications including peripheral neuropathy [21], diabetic retinopathy and cataracts [22–25], macrovascular disease [26] as well as platelet dysfunction [27,28]. However, very little attention has been paid to the potential adverse ramifications of chronic hyperglycemia driving polyol pathway overactivity in the brain.

We also observed that cord venous plasma (reflecting blood from placenta to fetus) sorbitol levels were ~7-fold higher than maternal plasma sorbitol levels, which is consistent with the presence of aldose reductase in the placenta [12] as well as recent evidence that sorbitol levels are high in fetal fluids during the first trimester [29]. Interestingly, unlike sorbitol, increased fructose levels were not observed in cord blood. Previous studies in sheep have suggested that the placenta has the capacity to generate sorbitol and fructose from glucose *in vivo* [30]. However, these data are inconsistent with data showing that sheep placenta expresses high levels of aldose reductase, but not sorbitol dehydrogenase [31,32]. Regardless, given that sorbitol dehydrogenase is known to be present in human fetal liver and brain [33], our findings raise the intriguing possibility that the sorbitol generated by the placenta could still be a substrate for fetal fructose production, which may have important ramifications for the developing fetal brain. Furthermore, because our samples were collected in the absence of labor, they may be a more accurate reflection of fetal exposure as it has been shown that stress from labor induces higher cord blood levels of glucose and fructose compared to levels obtained in the absence of labor [34]. This may explain why the levels of glucose and fructose we obtained in cord venous blood were lower than those obtained previously from cord blood in women after the onset of labor [35].

Whether pregnancy itself enhances the rate of flux through the polyol pathway remains unknown. It is noteworthy that we have obtained CSF and plasma from 2 obese non-pregnant women during large-volume (>30 ml) lumbar puncture for pseudotumor cerebri. There were no differences in the levels of sorbitol and fructose obtained from large volume lumbar puncture compared to the smaller volumes sampled in the pregnant women suggesting that any potential gradients between ventricular and spinal cord CSF are likely to be small. Furthermore, much like our pregnant women, there was a large discrepancy between their mean levels of fructose in CSF (0.14 ± 0.01 mmol/l) and plasma (0.005 ± 0.000). Thus, whether the flux through the polyol pathway observed in the current study is specifically linked to conditions associated with weight gain or diabetes requires further investigation.

The small sample size of this study may have limited our ability to detect differences in CSF fructose levels between lean, overweight/obese and diabetic pregnant individuals. There were no differences in fasting plasma or CSF glucose levels between the groups; however, there was a modest difference in HbA1C values (5.0–5.6% across all groups) which likely reflects increased post-prandial glucose levels in the obese and diabetic subjects who were more insulin resistant. During pregnancy, women are often more likely to adhere to stricter glycemic control and this may have contributed to the small differences in glycemic indices between our groups. Nevertheless, the significant correlation between CSF glucose levels and CSF fructose levels suggests that flux through the polyol pathway is present even in the absence of hyperglycemia and may be affected by circulating glucose levels in the normal range.

While every individual studied had higher CSF levels of fructose and sorbitol than plasma, the range of ratios observed was wide. This variation appeared to be driven in large part by variability in plasma fructose levels. However, the range of plasma levels of fructose in this study were similar to a previous report in non-pregnant diabetic and non-diabetic individuals using similar methodology [36]. Because peripheral fructose is metabolized primarily by the liver, it is possible that variable dietary habits as well as rates of hepatic gluconeogenesis among our pregnant subjects contribute to the observed variability in plasma fructose levels [37]. Finally, our CSF findings are only a surrogate for brain interstitial, and more importantly, intracellular levels. Because sorbitol is known to accumulate intracellularly due to its poor membrane permeability [38], the intracellular concentrations of sorbitol and fructose may exceed the CSF levels observed and may also explain why we did not observe a statistically significant correlation between CSF glucose and CSF sorbitol levels despite a clear correlation between CSF glucose and CSF fructose.

In summary, we provide evidence that the polyol pathway is likely to play a significant role in the conversion of glucose to sorbitol and fructose in the human brain. Furthermore, this pathway may also contribute to fetal exposure to sorbitol. The markedly higher CSF levels of sorbitol and fructose compared to maternal plasma suggest that CSF fructose may predominantly derive from local brain glucose metabolism via the polyol pathway rather than from BBB transport. These findings imply that fructose’s impact in the CNS may extend beyond its dietary consumption.
